# Comparison of the biopsy and cytobrush techniques for diagnosis of subclinical endometritis in mares

**DOI:** 10.1186/1477-7827-12-27

**Published:** 2014-04-04

**Authors:** Justyna Buczkowska, Roland Kozdrowski, Marcin Nowak, Andrzej Raś, Zdzisław Staroniewicz, Marta J Siemieniuch

**Affiliations:** 1 Faculty of Veterinary Medicine, Wrocław University of Environmental and Life Sciences, Wrocław, Poland; 2 Department and Clinic of Reproduction, Faculty of Veterinary Medicine, University of Warmia and Mazury, Olsztyn, Poland; 3 Department of Reproductive Immunology and Pathology, Institute of Animal Reproduction and Food Research of the Polish Academy of Sciences, Olsztyn, Poland

**Keywords:** Mare, Endometritis, Biopsy, Cytobrush, Cytology

## Abstract

**Background:**

Endometritis is a major cause of infertility in the mare. Therefore, the diagnosis of this disease is very important in veterinary practice. The objective of this study was to compare bacteriological and cytological results obtained from the mare uterus using biopsy (EB) and cytobrush (CB) techniques and relating these findings to the presence of polymorphonuclear cells (PMNs) in endometrial tissue as the gold standard for detection of endometritis. In particular, we tested the hypothesis that endometrial cytology and microbiology data obtained from material collected using the EB and CB techniques are similar, so that the CB technique could preferentially be used to detect subclinical endometritis in clinical practice.

**Methods:**

A total of 69 mares suspected of subclinical endometritis because of previous reproductive history and 15 maiden mares were enrolled in this study. Material collected from both EB and CB was smeared on sterile glass slides for cytological examinations and on culture media for microbiological examinations. Bacteriological cultures and cytological samples were classified as negative (no growth or mixed cultures of more than three microorganisms; <2% PMNs) or positive (pure growth of microorganisms; >2% PMNs) for endometritis.

**Results:**

Positive growth was observed in 43% of CB samples and in 54% of EB samples (difference not significant). The growth of β-hemolytic streptococci was always connected with positive cytology. This relationship was not observed for growth of *E. coli* or for non-pathogenic flora. The sensitivity of bacterial growth and cytology from EB was 0.63 and 0.73 respectively. The sensitivities of bacterial growth and cytology from CB were 0.50 and 0.71 respectively.

**Conclusion:**

Microbiological and cytological results obtained from CB are similar to those obtained from EB and based on these findings the CB technique may be recommended for collection of materials from the mare’s uterus in clinical practice.

## Background

Endometritis is a major cause of infertility in the mare [1-4]. Therefore the diagnosis of this disease is very important in veterinary practice [5]. Useful methods for the diagnosis of endometritis include clinical examination, transrectal palpation and ultrasonography of the reproductive tract, vaginal speculum examination, uterine culture, cytology and endometrial biopsy [2,3,5-7]. Mares suffering from subclinical endometritis usually do not show the typical symptoms associated with endometritis, i.e. accumulation of fluid in the uterus, and therefore the last three aforementioned tests to detect the disease are additionally required. Subclinical endometritis is influenced by the type of pathogen and the mare’s subsequent immunological response [3,7]. Endometritis is most commonly associated with aerobic bacteria [5]. However, isolation of bacteria does not necessarily prove the presence of endometritis nor does failure to isolate bacteria eliminate it [5,8,9]. It is believed that the combination of cytological and bacteriological diagnostics increases the ability to detect endometritis [5,7]. Moreover, the advantage of cytology is the ability to obtain results as early as the day of collection, while for bacteriological examination it takes 48-72 h from the time of sample collection [1,5,10-13]. Bourke and colleagues [14] compared the double guarded cotton swab (CS) and the uterine cytobrush (CB) techniques and showed that the latter was a superior method for collection of endometrial samples. More cases of endometritis are detected using CB than using the CS technique [7,13,15]. Moreover, higher sensitivity of the bacteriological test was reported after a biopsy had been examined [11].

To determine the relative importance of bacterial culture vs. cytology, it is helpful to have a gold standard for the presence or absence of disease against which the results can be compared. Many authors used as the gold standard the presence of neutrophil infiltration of the luminal epithelium and stratum compactum in uterine biopsies [11,16,17]. Endometrial biopsy is safe and useful, but not a particularly practical technique that allows an accurate assessment of the endometrium [8,18,19]. However, the histopathology is characterized by a long time waiting for the results and the special tools are needed for biopsy collection.

Previously, routine sampling of the mares uterus depended on the use of CS, while today the CB is used more frequently in the diagnosis of endometritis. Therefore, the objective of this study was to compare bacteriological and cytological results obtained from the mare uterus using both the biopsy (EB) and CB techniques and relating these findings to the presence of polymorphonuclear cells (PMNs) in endometrial tissue for the detection of endometritis. In particular, we tested the hypothesis that endometrial cytology and microbiology data obtained from material collected using the EB and CB techniques are similar, so that the CB technique could preferentially be used to detect subclinical endometritis in clinical practice.

## Methods

### Ethical approval for the use of animals

This study was approved by II Local Ethics Committee in Wrocław (Wrocław University of Environmental and Life Sciences, Poland). Reference number of approval: 43/2011, date: 18 April 2011.

### Animals

The study was conducted between February and September 2012 at a number of stud farms in Poland. The material was collected from 69 mares suspected of subclinical endometritis (aged 6-23 years) and from 15 maiden mares (young, aged 3-4 years, with no history of breeding). Mares suspected of subclinical endometritis had been bred three or more times unsuccessfully in the same breeding season, or had a history of one year of reproductive failure. Some of these mares had been treated with intrauterine antibiotic infusions in a previous breeding season and despite the treatment had not become pregnant. All mares were examined by transrectal palpation and ultrasonography for genital health and determination of cycle stage. None of the mares included in the study showed fluid in the uterus. Thirty-seven mares were in oestrus (exhibiting endometrial oedema and a dominant follicle) and 45 mares were in dioestrus (they showed no endometrial oedema and had a corpus luteum).

### Sample collection

Prior to sample collection, the mare’s tail was bandaged, the vulva and perineum were scrubbed with povidone-iodine and dried with a paper towel. An endometrial biopsy was collected using a sterilized biopsy punch (Equi-Vet, Kruuse; Denmark). Additionally, the biopsy punch was covered by a sanitary sleeve (Equi-Vet, Kruuse; Denmark). The instrument was passed through the vagina and cervix into the uterus with a sleeved and lubricated arm. After the forceps were placed in the uterine lumen, the arm was withdrawn from the vagina and inserted into the rectum to guide the forceps to the desired location. The uterine biopsy was taken from the base of the uterine horn.

Immediately after biopsy collection, the vulva and perineum were again cleaned and cellular material was collected from each mare using a commercially available CB (Cytology Brush; Minitube GmbH, Germany). The instrument was passed through the vagina and cervix into the uterus with a sleeved and lubricated arm. Cellular material was always collected from the base of the uterine horn opposite the site of biopsy collection by rotating the CB. The EB was always obtained before collection of cellular material similar to the protocol used by Nielsen [11].

Samples of EB and CB were immediately smeared on a sterile glass slide to obtain a cytological sample and afterwards were smeared onto culture media for microbiological examination. Finally, the end EB samples were fixed in formaldehyde for histopathological examination.

### Microbiology

All samples (from CB and EB) were smeared on a blood agar and also on Brain Heart Infusion Agar and on selective media: Eosin Methylene Blue Agar, Mannitol Salt Agar, Sabouraud Dextrose Agar. All culture media were obtained from Oxoid Ltd., Basingstoke; Hampshire, England. Culture media were incubated at 37°C in atmospheric air and after 24 hours growth of microorganisms was recorded. Culture plates with no growth were incubated and re-examined at 48 and 72 hours for the presence of bacteria or yeast. If >90% of the cultured colonies were of one species, they were considered to be a monoculture. Mixed cultures of more than three pathogens were considered as contamination [7,11]. A commercially available biochemical reaction system API was used, if further typing of cultures were needed. The common bacteria: β-hemolytic *Streptococcus, E. coli, Corynebacterium* spp. and *Pseudomonas* spp. were considered as equine uterine pathogens. Non-pathogenic bacteria were recorded only if isolated in pure culture [17].

### Cytology

After sampling and preparing the smears, the cytological specimens were fixed with Cytofix (Samko, Dobczyn; Poland) and stained using Diff Quick stain (Medion Diagnostics AG, Düdingen; Switzerland). The presence of PMNs was examined with light microscopy under oil immersion (1000× magnification). When PMN-cells represented more than 2% of all cells in the sample, it was considered positive for endometritis [7,20]. In each sample, 300 cells were counted.

### Histopathology

Endometrial biopsies were fixed in formalin and stained with haematoxylin and eosin. Biopsy specimens were evaluated by light microscopy for the presence of PMN-infiltration of the endometrial luminal epithelium and the stratum compactum. If one or more PMNs per five fields (400× magnification) occurred, the sample was considered as positive for endometritis [11,21].

### Statistical analysis

All comparisons were made by a Chi-square test and significance was set at *P* < 0.05. Microbiological and cytological results obtained from EB and CB were related to the histopathological presence of PMNs in the endometrium. Sensitivity, specificity, and positive and negative predictive value for these diagnostic tests were calculated. The sensitivity is the percentage of ill patients correctly diagnosed. The specificity is the percentage of healthy patients correctly diagnosed. The positive predictive values is the probability that positive results are really true, and the negative predictive values is the probability that negative results are really true. The sensitivity was calculated as the proportion of true positive results to true positive and false negative results. The specificity was calculated as the proportion of the true negative results to true negative and false positive results. The positive predictive value was calculated as the proportion of the true positive results to true positive and false positive results. The negative predictive value was calculated as the proportion of the true negative results to true negative and false negative results. True positive results are the number of mares with positive histopathology and positive cytology or microbiology (correctly identified). True negative results are the number of mares with negative histopathology and negative cytology or microbiology (correctly rejected). False positive results are the number of mares with positive cytology or microbiology but with negative histopathology (incorrectly identified) and false negative results are the number of mares with negative cytology or microbiology but with positive histopathology (incorrectly rejected).

## Results

Two cytological samples were not adequate for evaluation (hypocellularity). Consequently, cytological, bacteriological and histopathological specimens were examined and compared in 82 mares.

### Histopathological findings

A total of 56 (68.3%) mares had positive histopathology, including three maiden mares.

### Microbiological and cytological findings

**Figure 1:**
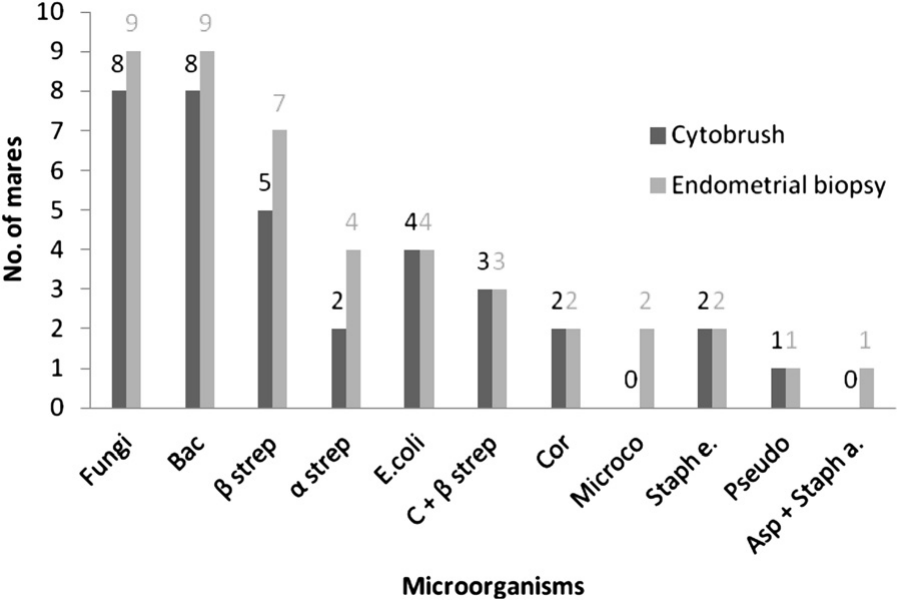
**Prevalence of microorganisms isolated from 82 mares using cytobrush (CB) and endometrial biopsy (EB) techniques.** Bac, Bacillus; β strep, β hemolytic Streptococcus; α strep, α hemolytic Streptococcus; C + β strep: number of cultures with Candida spp. and β hemolytic Streptococcus spp.; Cor, Corynebacterium spp., Microco, Micrococcus; Staph e., Staphylococcus epidermidis; Pseudo, Pseudomonas aeruginosa; Asp + Staph a.: number of cultures with Aspergillus spp. and Staphylococcus aureus isolated.

Results of uterine culture are shown in Figure 1. Positive growth from CB was obtained in 35 (43%) of all mares and in 44 (54%) from EB. We found no statistical differences in the number of positive cultures between CB and EB.

In 44 of 82 (54%) cytological smears obtained from CB and in 42/82 (51%) of cytological smears obtained from EB the percentage of PMNs was above 2%. This difference was not significant. Relationships between microorganisms and cytological findings are shown in Table 1. Both in CB and EB, 32% of cytologically positive mares also had positive cultures. The most common pathogenic bacteria cultured from EB and from CB were β-hemolytic streptococci, which was always associated with positive cytological findings. In 11% of samples collected using the CB technique, microbiology was positive but cytology was negative, but the isolated bacteria were *E. coli* or non-pathogenic species. In 22% of biopsy samples microbiology was positive but cytology was negative; *E. coli*, *Pseudomonas aeruginosa* and some non-pathogenic micro-organisms were cultured. Positive cytology was found in five maiden mares (from CB samples), and in three of these mares *Bacillus* was isolated.

**Table 1:** Relationship between microorganisms isolated and cytological findings

**Microorganisms**	**Cytobrush**			**Endometrial biopsy**		
	**Number of smears with negative cytology**	**Number of smears with positive cytology**	**Concordance between cytology and microbiology (%)**	**Number of smears with negative cytology**	**Number of smears with positive cytology**	**Concordance between cytology and microbiology (%)**
*β-hemolytic Streptococcus*	0	5	5/5 (100)	0	7	7/7 (100)
*α-hemolytic Streptococcus*	2	0	0/2 (0)	3	1	1/4 (25)
*E.coli*	2	2	2/4 (50)	3	1	1/4 (25)
*Fungi*	0	8	8/8 (100)	4	5	5/9 (56)
*Bacillus spp.*	4	4	4/8 (50)	6	3	3/9 (33)
*Corynebacterium spp.*	0	2	2/2 (100)	0	2	2/2 (100)
*Pseudomonas aeruginosa*	0	1	1/1 (100)	1	0	0/1 (0)
*Micrococcus spp.*	0	0	0	0	2	2/2 (100)
*Staphylococcus epidermidis*	1	1	1/2 (50)	1	1	1/2 (50)
*Candida + β hemolytic Streptococcus*	0	3	3/3 (100)	0	3	3/3 (100)
*Aspergillus + Staphylococcus aureus*	0	0	0	0	1	1/1 (100)

### Relationships of microbiological and cytological findings obtained from EB and CB to histopathology

Sensitivity, specificity, positive predictive value and negative predictive values of microbiological and cytological results obtained from EB and CB are presented in Table 2. Using the presence of PMN infiltration of the endometrial luminal epithelium and the stratum compactum as the gold standard for diagnosis of endometritis, the sensitivity of cytology from CB was 0.71 and from EB 0.73 and the sensitivity of microbiology from CB was 0.50 and from EB 0.63. When we combined results of cytological and bacteriological examinations, the sensitivity for both EB and CB increased (Table 3).

**Table 2:** Sensitivity, specificity, positive and negative predictive values data of cytological and microbiological examinations from cytobrush (CB) and endometrial biopsy (EB)

	**Histology positive**	**Histology negative**	**Sum**	**Sensitivity**	**Specificity**	**Positive predictive value**	**Negative predictive value**
*Microbiology of EB positive*	35	12	47	0.63	0.54	0.74	0.40
*Microbiology of EB negative*	21	14	35				
*Sum*	56	26	82				
*Microbiology of CB positive*	28	7	35	0.50	0.73	0.80	0.40
*Microbiology of CB negative*	28	19	47				
*Sum*	56	26	82				
*Cytology of EB positive*	41	1	42	0.73	0.96	0.98	0.63
*Cytology of EB negative*	15	25	40				
*Sum*	56	26	82				
*Cytology of CB positive*	40	4	44	0.71	0.85	0.91	0.58
*Cytology of CB negative*	16	22	38				
*Sum*	56	26	82				

**Table 3:** Sensitivity, specificity, positive and negative predictive values data of the combination of cytological and microbiological examinations from cytobrush (CB) and endometrial biopsy (EB)

	**Histology positive**	**Histology negative**	**Sum**	**Sensitivity**	**Specificity**	**Positive predictive value**	**Negative predictive value**
*Microbiology and cytology of EB positive*	51	12	63	0.91	0.54	0.81	0.74
*Microbiology and cytology of EB negative*	5	14	19				
*Sum*	56	26	82				
*Microbiology and cytology of CB positive*	48	8	56	0.86	0.69	0.86	0.69
*Microbiology and cytology of CB negative*	8	18	26				
*Sum*	56	26	82				

## Discussion

The types of microorganisms isolated from the uterus of mares in this study were similar to those reported in other studies [5,11,17,22,23]. However, in our study the most frequently isolated bacterium both from EB and CB was *Bacillus* which is considered to be non-pathogenic*.* Also in studies conducted by LeBlanc and colleagues [17] and Riddle and colleagues [5] non-pathogenic bacteria were frequently isolated. In our study, the growth of *Bacillus* was occasionally associated with positive cytology. Similarly, in studies conducted by LeBlanc and colleagues [17] and Riddle and colleagues [5], the isolation of *Bacillus* and other non-pathogenic species from the mare uterus was sometimes associated with positive cytology. The role of non-pathogenic bacteria isolated from the mare uterus is still unknown, however, some studies showed a possible association of these bacteria with decreased pregnancy rates [5]. We also rather frequently found fungi in materials collected using both CB and EB. This is not surprising if we take into consideration that most of the mares with growth of fungi had a history of frequently repeated intrauterine infusions of antibiotics in the previous breeding season. Similar to other studies, the most common pathogenic bacteria cultured from both EB and CB was β-hemolytic *Streptococcus*[5,11,23].

It should be noted that we found no statistical differences in the number of positive cultures between CB and EB or in positive cytology findings between CB and EB. In a study conducted by Overbeck and colleagues [7], bacteriological findings were not associated with positive cytology. In our study, isolation of β-hemolytic streptococci both in pure growth or in mixed growth with *Candida* was always associated with positive cytology. This relationship was also observed for the growth of *Corynebacterium* spp. but not for *E. coli*. Similarly in other studies, isolation of β-hemolytic streptococci was most commonly associated with positive cytology, and isolation of *E. coli* and other gram-negative bacteria was more likely not to associate with cytological evidence of inflammation [5,12]. It was shown in hysteroscopic examinations that *E. coli* can occur in local areas which can explain some of the failures to diagnose *E. coli*[17]. Walter and colleagues [15] found significant association between the number of colonies of β-hemolytic streptococci and the number of PMNs in smears, but this association was not observed for other microorganisms. LeBlanc and colleagues [17] suggested that the pathogenicity of *E. coli* appears to be different from pathogenicity of β-hemolytic *Streptococcus*, and consequently the uterine inflammatory response can vary with different microorganisms.

In our study, a total of 56 (68.3%) mares had positive histopathology, including three young mares. As indicated by the presence of PMNs in endometrial tissue the sensitivity of cytology obtained from EB and CB was superior to the sensitivity of microbiological examinations from both EB and CB. These results showed that cytological examination is more accurate than microbiology for diagnosis of inflammation of the endometrium. Also in another study, positive cytology was twice as common as a positive culture [5]. Additionally, cytological examinations of samples collected using the EB or CB techniques had higher positive predictive value than bacteriological examination with both sampling methods. This demonstrates that cytological examination is a more accurate method for diagnosis of endometritis than bacteriology and any positive cytology should be considered with high probability as indicative of endometritis. However, we must remember that this technique does not provide information about a cause of the inflammation.

The sensitivity of bacterial growth from EB presented in our study was lower than that estimated by Nielsen [11]. In contrast, the sensitivity of microbiology and cytology obtained both from EB and CB was much higher than in the study conducted by Overbeck and colleagues [7]. Additionally, in our study the specificity of bacterial growth from CB was higher than from EB, most likely because of the reduced risk of contamination of the sample collected using CB.

When we combined the results of cytological and bacteriological examinations, the sensitivity increased. Overbeck and colleagues [7] also showed that the combination of cytological and bacteriological examination of endometrial samples collected using CB might be the most promising approach for diagnosing endometritis.

## Conclusions

The long time between sampling and laboratory results is a practical disadvantage of the biopsy approach while results of cytology are rapid. A biopsy is not always possible, but CB is a quick, relatively inexpensive and safe technique, easy to obtain under field conditions and enables the diagnosis of subclinical endometritis. Consequently, based on our results we can recommend the CB technique for diagnosis of subclinical endometritis in the mare, when the samples are collected during oestrus or dioestrus and examined both cytologically and microbiologically.

## Abbreviations

CB: Cytobrush; EB: Endometrial biopsy; PMNs: Polymorphonuclear cells.

## Competing interests

The authors declare that they have no competing interests.

## Authors’ contributions

JB contributed to sample collection, cytological analysis, analysis of the results and writing the manuscript. RK contributed in conceiving the study, sample collection, cytological analysis, analysis of the results and writing the manuscript. MN contributed by histopathological examination. AR contributed in conceiving the study and sample collection. ZS contributed by microbiological examination, and MJS contributed in conceiving the study and sample collection. All authors read and approved the final manuscript.
